# KIR 2D (L1, L3, L4, S4) and KIR 3DL1 protein expression in non-small cell lung cancer

**DOI:** 10.18632/oncotarget.13486

**Published:** 2016-11-21

**Authors:** Yayi He, Paul A. Bunn, Caicun Zhou, Dan Chan

**Affiliations:** ^1^ Department of Oncology, Shanghai Pulmonary Hospital, Tongji University Medical School Cancer Institute, Tongji University School of Medicine, Shanghai, People's Republic of China; ^2^ Department of Medicine, Division of Medical Oncology, University of Colorado Anschutz Medical Campus, Aurora, CO, USA

**Keywords:** KIR 2D (L1, L3, L4, S4), KIR 3DL1, NSCLC, Pathology Section

## Abstract

**Background:**

Nature killer (NK) cells are the immune system's first line of defense against both viral infections and tumors. Killer cell immunoglobulin-like receptors (KIRs) are associated with susceptibility to different types of cancers. We investigated KIR 2D (L1, L3, L4, S4) and KIR 3DL1 protein expression and their association with survival in non-small cell lung cancer (NSCLC).

**Methods:**

The expression of KIR 2D (L1, L3, L4, S4) (BC032422/ ADQ31987/ NP_002246/ NP_036446, ABCAM) and KIR 3DL1 (AA 1-444, ABCAM) protein was assessed by immunohistochemistry (IHC) in 62 NSCLC patients.

**Results:**

KIR 2D (L1, L3, L4, S4) and KIR 3DL1 were expressed both on NSCLC tumor cells and tumor infiltrating lymphocytes (TILs). Fourteen samples (22.6%) stained positive for KIR 2D (L1, L3, L4, S4) on the tumor cells, and 10 (16.1%) had positive expression on the TILs. Thirty-three samples (53.2%) stained positive for KIR 3DL1 on the tumor cells, and 31 (50.0%) had positive expression on the TILs. Patients with negative KIR 2D (L1, L3, L4, S4) expression on tumor cells or TILs had longer overall survival (OS) than patients who are KIR 2D (L1, L3, L4, S4) positive on tumor cells (40.70 weeks, 95% CI 24.76-56.65 *vs.* 7.10 weeks, 95% CI 0.00-19.38, *P* = 0.014) or TILs (40.70 weeks, 95% CI 24.05-57.35 *vs.* 3.90 weeks, 95% CI 0.00-9.17, *P* < 0.001). Likewise, longer OS was significantly correlated with negative expression of KIR 3DL1 on tumor cells (62.30 weeks, 95% CI 0.00-177.37 *vs.* 13.10 weeks, 95% CI 3.42-22.78, *P* < 0.001) or TILs (62.30 weeks, 95% CI 0.00-152.05 *vs.* 12.10 weeks, 95% CI 2.61-21.59, *P* < 0.001). Cox regression analysis showed that KIR 2D (L1, L3, L4, S4) on TILs was correlated with OS (*P* = 0.032, Odds Ratio 2.628 95%CI 1.089-6.340).

**Conclusions:**

KIR 2D (L1, L3, L4, S4) and KIR 3DL1 expression was correlated with poor prognosis in NSCLC patients.

## INTRODUCTION

Lung cancer is the primary cause of cancer death worldwide [1]. Chemotherapy, the standard first line therapy for advanced stage lung cancer, has a poor prognosis. Targeted therapy offers a more promising outcome in advanced non-small cell lung cancer (NSCLC) [2–4], but only patients who harbor driving mutations such as epidermal growth factor receptor (EGFR) potentially benefit [5, 6]. Immunotherapy can reverse tumor immune escape [7]. The inhibition of checkpoints, such as cytotoxic T lymphocyte antigen-4 (CTLA-4), programmed death-1 (PD-1), and programmed death ligand-1 (PD-L1) may produce more positive outcomes in lung cancer patients [8–13].

Human leukocyte antigen-I (HLA-I) and related killer cell immunoglobulin like receptors (KIRs) molecules are additional important molecules which could facilitate immune escape in cancer [14]. The KIRs are a family of receptors encoded by 14 polymorphic genes (KIR2DL1-5, KIR3DL1-3, KIR2DS1-5, KIR3DS1), seven of which are inhibitory and seven of which are activating [15]. Upregulating the specific KIR ligand either on the tumor or on the tumor infiltrating lymphocyte (TILs) could inhibit the anti-tumor immune reaction [16, 17]. Currently, little is understood about the mechanisms of KIRs in the lung cancer immune system.

In this study, we investigated KIR 2D (L1, L3, L4, S4) and KIR 3DL1 protein expression in NSCLC patient tumor tissues by immunohistochemistry (IHC) and analyzed the correlation between KIR 2D (L1, L3, L4, S4), KIR 3DL1 and clinical pathological characteristics. We also conducted survival analysis in NSCLC patients.

## RESULTS

### Patient characteristics

NSCLC patient tissues were obtained from 62 patients from Dan Lab (University of Colorado) between January 2004 and November 2008. Among them, 30 (48.4%) were male and 32 (51.6%) were female. The median age was 63 years old. Thirteen (21.0%) were never smokers. Twelve patients (19.4%) were stage I or II, and 50 (80.6%) were stage III or IV. Forty-two patients (67.7%) had adenocarcinoma, and 16 (25.8%) had squamous cell carcinoma (SCC) (Table 1).

**Table 1:** Patient Characteristics (*n* = 62)

Characteristic	Total
**Age, median**	63
<70, *n* (%)	44 (71.0%)
≥70, *n* (%)	18 (29.0%)
**Gender,*n* (%)**	
Male	30 (48.4%)
Female	32 (51.6%)
**Smoking status,*n* (%)**	
Non-smoker	13 (21.0%)
Smoker	49 (79.0%)
**Lung cancer staging,*n* (%)**	
I-II	12 (19.4%)
III-IV	50 (80.6%)
**Pathology,*n* (%)**	
SCC	16 (25.8%)
Adenocarcinoma	42 (67.7%)
NSCLC NOS/Mixed	4 (6.5%)

### Characterization of KIR 2D (L1, L3, L4, S4) and KIR 3DL1 in lung cancer and their association with clinical pathological factors

**Figure 1:**
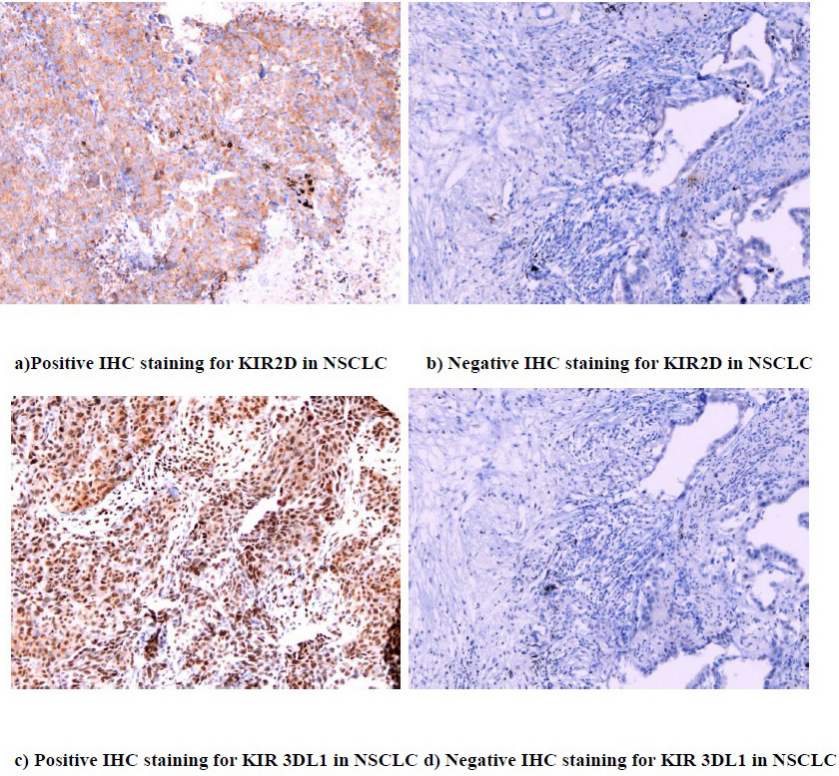
IHC staining for KIR 2D (L1, L3, L4, S4) and KIR 3DL1 (20X)

KIR 2D (L1, L3, L4, S4) and KIR 3DL1 were expressed on both lung cancer cells and TILs. Fourteen samples (22.6%) stained positive for KIR 2D (L1, L3, L4, S4) on the tumor cells, and 10 (16.1%) had positive expression on the TILs. Thirty-three samples (53.2%) stained positive for KIR 3DL1 on the tumor cells, and 31 (50.0%) had positive expression on the TILs (Figure 1). High expression of KIR2D on tumor cells was significantly correlated with higher expression of KIR2D on TILs (*P* < 0.001), KIR 3DL1 on tumor cells (*P* = 0.001) and KIR 3DL1 on TILs (*P* < 0.001). The same relationship was also found between KIR2D on TILs and KIR 3DL1 on tumor cells (*P* = 0.001), KIR2D on TILs and KIR 3DL1 on TILs (*P* < 0.001), and KIR 3DL1 on tumor cells and KIR 3DL1 on TILs (*P* < 0.001) (Table S1). Neither the expression of KIR 2D (L1, L3, L4, S4) nor KIR 3DL1 correlated significantly with clinical pathological factors (Table S2).

### Univariate and multivariate logistic analysis for predicting KIR 2D (L1, L3, L4, S4) and KIR 3DL1 expression

With the Cox regression model, no variable (age, gender, smoking history, lung cancer stage, pathology) predicted KIR 2D (L1, L3, L4, S4) or KIR 3DL1 expression on tumor cells or TILs (Table S3, S4).

### Association between KIR 2D (L1, L3, L4, S4), KIR 3DL1 and OS in lung cancer patients

**Figure 2:**
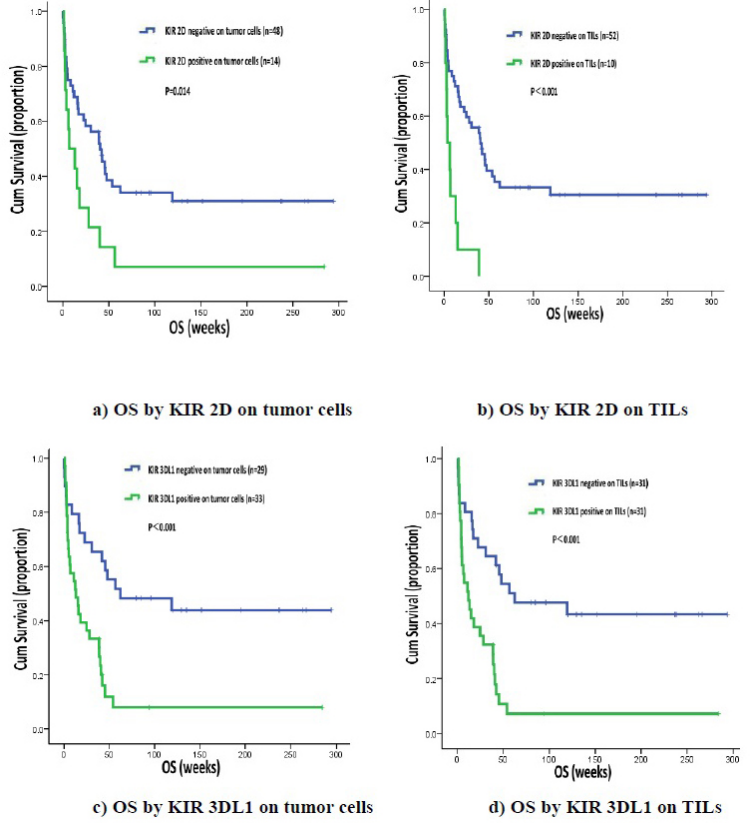
OS by KIR 2D (L1, L3, L4, S4) and KIR 3DL1

We found that patients who were KIR 2D (L1, L3, L4, S4) negative on tumor cells or TILs had longer OS than patients who are KIR 2D (L1, L3, L4, S4) positive on tumor cells (40.70 weeks, 95% CI 24.76-56.65 *vs*. 7.10 weeks, 95% CI 0.00-19.38, *P* = 0.014) or TILs (40.70 weeks, 95% CI 24.05-57.35 *vs*. 3.90 weeks, 95% CI 0.00-9.17, *P* < 0.001). Likewise, OS was significantly longer in patients not expressing KIR 3DL1 on tumor cells (62.30 weeks, 95% CI 0.00-177.37 *vs*. 13.10 weeks, 95% CI 3.42-22.78, *P* < 0.001) or TILs (62.30 weeks, 95% CI 0.00-152.05 *vs*. 12.10 weeks, 95% CI 2.61-21.59, *P* < 0.001) (Figure 2).

### Cox regression analysis of OS

Cox regression analysis showed that KIR 2D (L1, L3, L4, S4) on TILs was correlated with OS (*P* = 0.032, Odds Ratio 2.628 95%CI 1.089-6.340) (Table 2).

**Table 2:** COX regression analysis of OS

	Univariate			Multivariate		
Variables	Odds Ratio	95% CI	*P*	Odds Ratio	95% CI	*P*
Age (<70 vs. ≥70)	1.260	0.661-2.403	0.483			
Gender (Female vs. Male)	0.837	0.465-1.506	0.552			
Smoking status (Non-smoker vs. Smoker)	1.115	0.552-2.254	0.761			
Stage (I-II vs. III-IV)	1.772	0.768-3.861	0.187			
Pathology (NSCLC vs. SCLC)	0.677	0.354-1.293	0.237			
KIR 2D on TILs (Negative vs. Positive)	2.219	1.151-4.277	**0.017**	1.020	0.463-2.246	0.961
KIR 2D on tumor cells (Negative vs. Positive)	4.273	1.976-9.239	**<0.001**	2.628	1.089-6.340	**0.032**
KIR 3DL1 on tumor cells (Negative vs. Positive)	3.107	1.625-5.939	**0.001**	1.426	0.185-10.985	0.734
KIR 3DL1 on TILs (Negative vs. Positive)	3.187	1.689-6.012	**<0.001**	1.857	0.244-14.133	0.550

## DISCUSSION

In this study, we investigated KIR 2D (L1, L3, L4, S4) and KIR 3DL1 protein expression in NSCLC tumor tissues and analyzed the correlation between KIR 2D (L1, L3, L4, S4), KIR 3DL1 and clinical pathological characteristics. We also conducted survival analysis in NSCLC patients.

Natural killer (NK) cells perform critical functions in the innate immune response. They both control viral infection and eliminate early stage cancer [18–21]. It remains unknown whether NK cells are directly anti metastatic or rather suppress antitumor immunity [22–24]. NK cells expressing NK cell receptors (NKRs) can release cytokines that activate antitumor effector cells to inhibit tumor cells. The receptors expressed by NK cells, CD94: NKG2 heterodimers and KIRs, could regulate NK cell activity [14].

It is possible that KIRs may modulate NK cell and T lymphocytes function, effecting cancer immune response. A correlation between KIR/HLA compound genotypes, viral infections, chronic inflammatory diseases, and autoimmune diseases has been reported [25]. In addition, KIR/HLA compound genotype is associated with susceptibility to leukemia, cervical neoplasia, melanoma, and Epstein-Barr virus (EBV)-associated nasopharyngeal carcinoma (NPC) [18–21, 26–33]. It has been reported that the KIR 2DL2 phenotype frequency in leukemic patients was significantly higher than that in the general population. Moreover, almost all inhibitory KIRs were highly expressed in leukemia patients. KIR 3DL1 was expressed in almost all leukemia patients. In leukemia, KIR phenotype induced immune escape from NK cells [27]. KIRs also increased the risk of cervical intraepithelial neoplasia [28]. Combinations of KIRs and HLA affect the progression of cervical neoplasia. The presence of the activating receptor KIR 3DS1 resulted in increased risk of cervical neoplasia [29]. KIRs in EBV-associated NPC patients tend to more activating than those in healthy subjects [34]. Besson et al. found an association between Hodgkin's lymphoma and certain KIR alleles in strong linkage disequilibrium [31]. KIR 2DL2 receptors were expressed at higher levels in breast cancer patients relative to healthy controls. KIR 2DL2 in combination with the HLA-C heterozygote ligand (C1/C2) could increase susceptibility to breast cancer [19]. We detected KIR 2D (L1, L3, L4, S4) and KIR 3DL1 on both tumor cells and TILs in NSCLC, but found no correlation with clinical characteristics.

KIRs have already been reported on tumor-specific CTLs in malignant tumor [26, 35]. Functionally, those CTLs exhibited a low level of lytic activity against autologous tumor cells, which was dramatically increased upon KIR blockade with the specific antibody. In contrast to the constitutive and stable KIR expression in mature NK cells, the induction of a particular KIR repertoire in T cells under specific conditions might be one of the events leading to the lethargy of the immune effector cells [36]. Engagement of inhibitory KIR with the ubiquitous MHC-I molecules on the surface of most cells establishes NK cell tolerance toward normal cells. Upon interaction with MHC-I ligands on the target cells, KIR recruit protein tyrosine phosphatases to the plasma membrane, which counteract activating receptor signals to inhibit cytotoxicity and cytokine production [37]. Because inhibitory KIRs could regulate NK cell activation, therapeutic strategies which inhibit KIRs could combat cancer by improving NK cells' activity. KIR blockade may be a therapeutically viable option to boost NK cell mediated cytotoxicity responses in cancer patients [38]. We conducted a survival analysis and found that positive KIR 2D (L1, L3, L4, S4) and KIR 3DL1 expression on tumors and TILs was correlated with poor prognosis in NSCLC patients.

This study is limited in that it is retrospective and assessed a relatively small number of patients. We are going to expand our patient sample set and conduct a larger prospective study. Additionally, previous research had not established the cutoff for determination of positive KIR 2D (L1, L3, L4, S4) or KIR 3DL1 protein expression in lung cancer by IHC, nor was one provided by Abcam. Therefore, we decided the cutoff that we found best for predicting OS.

In this study, we analyzed the relationship between KIR 2D (L1, L3, L4, S4), KIR 3DL1 expression and clinical characteristics. We also examined the association between expression of KIR 2D (L1, L3, L4, S4), KIR 3DL1 and OS. We have demonstrated that KIR 2D (L1, L3, L4, S4) are KIR 3DL1 were highly expression on NSCLC tumor cells and TILs. Furthermore, positive KIR 2D (L1, L3, L4, S4) or KIR 3DL1 expression on tumor cells or TILs was correlated with poor prognosis in NSCLC patients. Additional studies are needed to analyze the mechanisms of the of KIR/HLA interactions that affect NK cells in lung cancer.

## MATERIALS AND METHODS

### Patients

Primary tumor specimens were obtained from 62 NSCLC patients from Dan Lab (University of Colorado) between January 2004 and November 2008. All patients were newly diagnosed with NSCLC. The patients had not undergone radiation or chemotherapy before the biopsy. Lung cancer stages were categorized by the 7^th^ edition International Association for the Study of Lung Cancer (IASLC) TNM staging system. The protocol was approved by the Shanghai Pulmonary Hospital, Tongji University. All participants were competent to provide their consent.

### IHC for KIR 2D (L1, L3, L4, S4) and KIR 3DL1

Paraffin tissue sections were baked in drying oven at 60°C for 1 hour. Slides were labeled and put in a Benchmark XT® (Ventana Medical Systems, Inc) autostainer. After treating slides with standard cell conditioning 1 for 60 minutes, KIR antibodies (KIR 2DL1+KIR 2DL3+KIR 2DL4+KIR 2DS4, BC032422/ ADQ31987/ NP_002246/ NP_036446, 1:75, ABCAM) (KIR 3DL1, AA 1-444, 1:1500, ABCAM) were applied, and the slides were incubated at 37°C for 1 hour. UltraView DAB detection and amplification kit was used. Slides were counterstained with hematoxylin for 4 minutes then post-counterstained with bluing agent for 4 minutes. Slides were washed then dehydrated in 70% to 100% reagent alcohol and xylenes baths before application of coverslips. We used human tonsil as the positive control for KIR 2D (L1, L3, L4, S4) and KIR 3DL1.

### Determination of KIR 2D (L1, L3, L4, S4) and KIR 3DL1 IHC cutoff

All IHC results were independently checked by two pathologists. Staining was evaluated using the H score system [39]. We defined KIR 2D (L1, L3, L4, S4) positivity as an H score greater than 200 for tumor cells and greater than 210 for TILs, respectively. We chose to define KIR 3DL1 positivity on tumor cells and TILs as greater than 200 because that cut off best predicted OS.

### Statistical Analysis

We performed statistical analysis by SPSS statistical software package (version 17.0; SPSS, Inc.; Chicago, IL). Chi-square tests were used to analyze correlation between KIR 2D (L1, L3, L4, S4) and KIR 3DL1 protein expression and clinical pathological variables. The odds ratios for positive KIR 2D (L1, L3, L4, S4) and KIR 3DL1 expression were calculated by a logistic regression model for factors including age, gender, smoking status, lung cancer stage and pathology. The survival curves were estimated by the Kaplan-Meier method. Univariate and multivariate analysis was performed using the Cox regression model to investigate the relationships between the correlative factors of age, gender, smoking status, pathology, lung cancer stage, KIR 2D (L1, L3, L4, S4) on tumor cells and TILs, KIR 3DL1 on tumor cells and TILs and OS. All statistics were 2-sided and statistical significance was defined as *P*<0.05.

## SUPPLEMENTARY MATERIALS


