# Supplementary material for: KIR 2D (L1, L3, L4, S4) and KIR 3DL1 protein expression in non-small cell lung cancer
Source: Oncotarget. 2016 Nov 21;7(50):82104–11. doi: 10.18632/oncotarget.13486 (PMC5347678; doi:10.18632/oncotarget.13486)
Supplement: Supplementary file 1 [file oncotarget-07-82104-s001.pdf]

KIR 2D (L1, L3, L4, S4) and KIR 3DL1 protein expression in non-small cell lung cancer

Supplementary Material

Table S1. The correlation between KIR 2D (L1, L3, L4, S4) and KIR 3DL1

	KIR2D on TILs			KIR 3DL1 on tumor cells			KIR 3DL1 on TILs		
	negative	positive	p	negative	positive	p	negative	positive	p
**KIR2D on tumor cells**									
**negative**	47(97.9%)	1(2.1%)	0.000	28(58.3%)	20(41.7%)	0.001	30(2.5%)	18(37.5%)	0.000
**positive**	5(35.7%)	9(64.3%)		1(7.1%)	13(92.9%)		1(7.1%)	13(92.9%)	
**KIR2D on TILs**									
**negative**				29(55.8%)	23(44.2%)	0.001	31(59.6%)	21(40.4%)	0.000
**positive**				0(0.0%)	10(100.0%)		0(0.0%)	10(100.0%)	
**KIR 3DL1 on tumor cells**									
**negative**							29(100.0%)	0(0.0%)	0.000
**positive**							2(6.1%)	31(93.9%)	

Table S2. Relationships between KIR 2D (L1, L3, L4, S4), KIR 3DL1 and clinical data

	KIR2D on tumor cells			KIR2D on TILs			KIR 3DL1 on tumor cells			KIR 3DL1 on TILs		
	Negative	Positive	P	Negative	Positive	P	Negative	Positive	P	Negative	Positive	P
**Age, n (%)**												
<70	33 (75.0%)	11 (25.0%)	0.739	37 (84.1%)	7 (15.9%)	1.000	20 (45.5%)	24 (54.5%)	0.785	22 (50.0%)	22 (50.0%)	1.000
≥70	15 (83.3%)	3 (16.7%)		15 (83.3%)	3 (16.7%)		9 (50.0%)	9 (50.0%)		9 (50.0%)	9 (50.0%)	
**Gender, n (%)**												
Female	24 (75.0%)	8 (25.0%)	0.764	25 (78.1%)	7 (21.9%)	0.304	13 (40.6%)	19 (59.4%)	0.445	14 (43.8%)	18 (56.3%)	0.446
Male	24	6		27	3		16	14		17	13	

	(80.0%)	(20.0%)		(90.0%)	(10.0%)		(53.3%)	(46.7%)		(56.7%)	(43.3%)	
**Smoking status, n (%)**												
Non-smoker	38 (77.6%)	11 (22.4%)	1.000	41 (83.7%)	8 (16.3%)	1.000	22 (44.9%)	27 (55.1%)	0.756	24 (49.0%)	25 (51.0%)	1.000
Smoker	10 (76.9%)	3 (23.1%)		11 (84.6%)	2 (15.4%)		7 (53.8%)	6 (46.2%)		7 (53.8%)	6 (46.2%)	
**Lung cancer staging, n (%)**												
Stage I-II	11 (91.7%)	1 (8.3%)	0.267	12 (100%)	0 (0.0%)	0.186	8 (66.7%)	4 (33.3%)	0.197	8 (66.7%)	4 (33.3%)	0.335
Stage III-IV	37 (74.0%)	13 (26.0%)		40 (80.0%)	10 (20.0%)		21 (42.0%)	29 (58.0%)		23 (46.0%)	27 (54.0%)	
**Pathology, n (%)**												
Adenocarcinoma	32 (76.2%)	10 (23.8%)	1.000	33 (78.6%)	9 (21.4%)	0.146	19 (45.2%)	23 (54.8%)	0.789	20 (47.6%)	22 (52.4%)	0.786
Non-adenocarcinoma	16 (80.0%)	4 (20.0%)		19 (95.0%)	1 (5.0%)		10 (50.0%)	10 (50.0%)		11 (55.0%)	9 (45.0%)	

Table S3. Univariate analysis for prediction of KIR 2D (L1, L3, L4, S4) expression on tumor cells and TILs

	Tumor cells			TILs		
**Variable**	**Odds Ratio**	**95% CI**	**P**	**Odds Ratio**	**95% CI**	**P**
Age (<70 vs.≥70)	0.600	0.146-2.470	0.479	1.057	0.241-4.642	0.941

Gender (Female vs. Male)	0.750	0.226-2.491	0.639	0.397	0.092-1.705	0.214
Smoking status (Non-smoker vs. Smoker)	1.036	0.242-4.437	0.962	0.932	0.173-5.032	0.935
Stage (I-II vs. III-IV)	3.865	0.454-32.929	0.216	$4.0*10^8$	0.000-?	0.999
Pathology (Adenocarcinoma vs. Non-adenocarcinoma)	0.800	0.217-2.952	0.738	0.193	0.023-1.643	0.132

Table S4. Univariate and multivariate analysis for prediction of KIR 3DL1 expression on tumor cells and TILs

	**Tumor cells**			**TILs**		
**Variable**	**Odds Ratio**	**95% CI**	**P**	**Odds Ratio**	**95% CI**	**P**
Age (<70 vs.≥70)	0.833	0.278-2.499	0.745	1.000	0.334-2.994	1.000
Gender (Female vs. Male)	0.599	0.219-1.637	0.318	0.595	0.218-1.624	0.311
Smoking status (Non-smoker vs. Smoker)	0.698	0.205-2.383	0.566	0.823	0.242-2.804	0.755
Stage (I-II vs. III-IV)	2.762	0.734-10.392	0.133	2.348	0.625-8.814	0.206
Pathology (Adenocarcinoma vs. Non-adenocarcinoma)	0.826	0.284-2.400	0.726	0.744	0.255-2.166	0.587
